# WSN-Based Space Charge Density Measurement System

**DOI:** 10.1371/journal.pone.0169034

**Published:** 2017-01-04

**Authors:** Dawei Deng, Haiwen Yuan, Jianxun Lv, Yong Ju

**Affiliations:** School of Automation Science and Electrical Engineering, Beihang University, Beijing, China; West Virginia University, UNITED STATES

## Abstract

It is generally acknowledged that high voltage direct current (HVDC) transmission line endures the drawback of large area, because of which the utilization of cable for space charge density monitoring system is of inconvenience. Compared with the traditional communication network, wireless sensor network (WSN) shows advantages in small volume, high flexibility and strong self-organization, thereby presenting great potential in solving the problem. Additionally, WSN is more suitable for the construction of distributed space charge density monitoring system as it has longer distance and higher mobility. A distributed wireless system is designed for collecting and monitoring the space charge density under HVDC transmission lines, which has been widely applied in both Chinese state grid HVDC test base and power transmission projects. Experimental results of the measuring system demonstrated its adaptability in the complex electromagnetic environment under the transmission lines and the ability in realizing accurate, flexible, and stable demands for the measurement of space charge density.

## Introduction

High voltage direct current (HVDC) transmission system is widely applied for its economical, technical, and environmental advantages in both long distance and bulk power transmission. However, the consequent electromagnetic environmental problem has caused wide public concern as well as huge psychological pressure. The detection of electromagnetic environment plays an important role in the construction of high voltage transmission line. The use of high voltage transmission lines can cause the increase of the space charge density of the conductors, which may exert psychological and physiological impacts on human body [1–3]. The parameters of electromagnetic environment under HVDC transmission lines mainly consists of space charge density, ion current density, magnetic induction intensity, audible noise and radio interference, among which the space charge density is believed to be an important parameter for evaluation of electromagnetic environment that directly affects human body. Space charge density produced by electrical charges resides on both conductive surfaces and space charges. In order to improve the measuring equipment design and limit the space charge density to satisfy the electromagnetic environmental standard, the development of an accurate and efficient measuring method and system is extremely urgent [4,5].

The area of HVDC transmission line is so large that makes it inconvenient to use cable for space charge density monitoring system, while the situation can be improved by Wireless Sensor Network (WSN). WSN is a complex network system and integrates data acquisition, processing and transmission, which consists of sensor module, wireless communication module, data processing module, power supply module and so on [6–9]. Compared with the traditional communication network, WSN has the advantages of small volume, high flexibility, strong self-organization and so on [10–13]. It is more suitable for the construction of distributed space charge density monitoring system as it has longer distance and higher mobility [14–18].

The main purpose of this paper is to design a wireless sensor network for accurately and flexibly measuring the space charge under HVDC transmission lines. This paper is organized as follows. In Section 2, the measurement principle of space charge density measurement system is described. In Section 3, the wireless node connected with the sensor is proposed. In Section 4, the design of wireless measurement system is elaborated based on space charge density measuring system and wireless node. Finally, experimental data derived from HVDC space charge density measurement system is carefully analyzed. A discussion and conclusion section ends this paper [19–23].

## Space Charge Measurement System Framework

Space charge moves directionally between HVDC power transmission line and the earth. Due to the difference between positive and negative electrode polarity, the formation mechanism of space charge and moving direction are of slight difference. The corona discharge of the positive electrode leads to the ionization of oxygen and nitrogen in air. Under the electric field force, the newly generated electrons is capable of obtaining kinetic energy, re-colliding with oxygen and nitrogen and therefore generating more electrons, which would contribute to the rapid development of ionization and formation of more positive ions. The positive ions move from the conductor to the earth with the existence of electric field. The corona discharge of the negative wire is a reflection of ionization of oxygen and water vapor in air.

**Fig 1:**
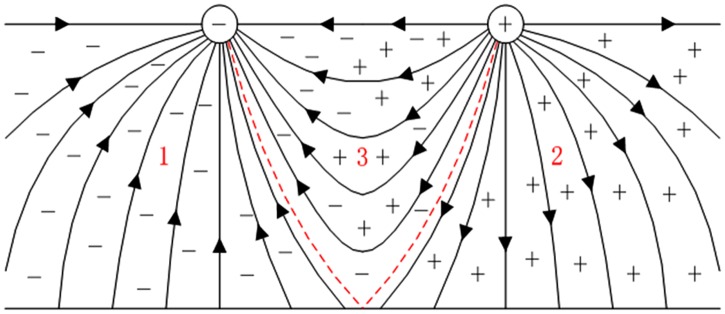
Distribution of space charge around HVDC transmission line.

In the corona layer, the electrons are subjected to the repulsive force of electric field generated by the conducting wire and move away from the conducting wire. The electric field density is weak outside the corona layer. The electron on the corona layer is attached to the neutral molecule to form a negative ion, which is subjected to the electric field force and moves away from the wire to the ground motion, during which both the motion of ions and charge is a dynamic process. The distribution of space charge can be regarded as quasi steady state under line operation condition. Macroscopically speaking, the ionization region is thin and can be ignored. Herein, three regions of the transmission line in the surrounding space can be formed, as presented in Fig 1: region 1 and 2 are mainly negative and positive ions, respectively; while region 3 is a mixture of both negative and positive ions.

**Fig 2:**
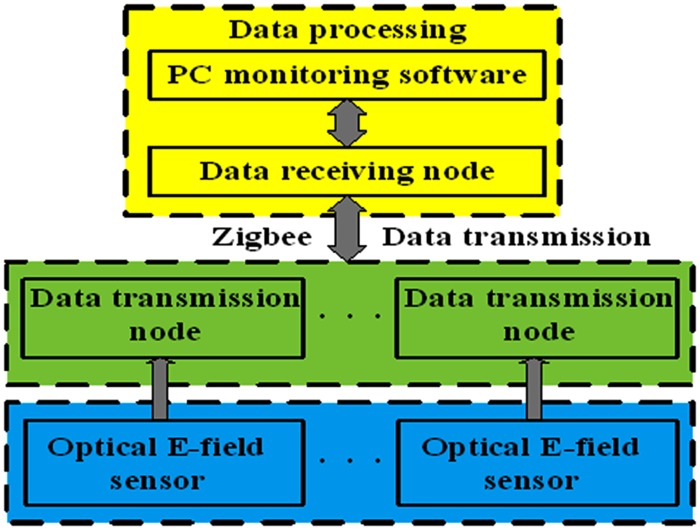
Framework of the space charge measurement.

The space charge under HVDC transmission lines is measured at different positions through many wireless sensors, these measured data can show the distribution of space charge. The framework of the space charge measurement is shown in Fig 2. The whole measuring system is composed of three parts: signal sensing, data transmission and data processing. As for signal sensing, a large number of space charge sensors are arranged to convert the induced space charge to the measured analog voltage signal by a certain relationship, followed by the output of space charge. A lot of data transmission nodes are used in data transmission, which can be converted into digital voltage signal by Zigbee sampling and convert the analog voltage signal into digital voltage signal by AD. Data receiving nodes are used for receiving and integrating the radio frequency signal which is transmitted by the data transmitting area. The host computer monitoring software is responsible for monitoring the space charge data. The value of the space charge is displayed on the monitor and stored in the database. As a result, the distribution of the space charge under the transmission lines can be achieved through the usage of multiple sensors system.

## Design of Charge measurement instrument and Wireless Node

### Charge measuring instrument

The charged ions in air can be sucked into the measuring device under the action of fan. Charge will be adsorbed on the filter while charged ion is adsorbed on filter material. By acquiring and measuring the static charge on filter material and the certain time through the filter, the gas volume can be calculated at a location of certain space charge density. Finally, the space charge density of the region is used as the space charge density of the space in a certain period of time.

**Fig 3:**
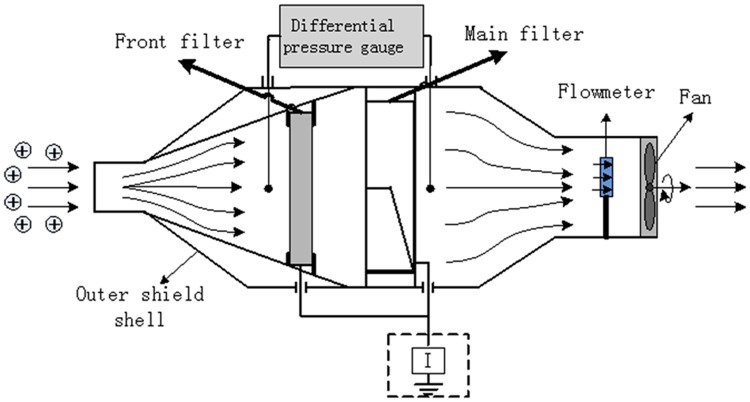
Structure of space charge density measurement instrument.

The space charge sensor mainly includes outer shielding shell, front filter, main filter, flow meter, differential pressure gauge, fan and measuring circuit. Outer shielding shell is utilized to prevent the sensor from being influenced by external electromagnetic field. Front filter setting can improve the life of main filter and filtering efficiency. Heat flow meter is used to calculate the volume of gas in a certain period of time, which can meet the demand of the actual engineering. Differential pressure gauge can detect the failure and ensure the stable operation of device. Measuring circuit mainly includes charge measuring circuit and flow measuring circuit. The structure of the space charge sensor is shown in Fig 3.

The calculating formula of space charge density is shown as formula 1.

$$\rho =\frac{Q}{V}=\frac{\int_{0}^{t}I(t)dt}{tM_{0}}$$ (1)

where *ρ* is the space charge density, *Q* represents the adsorption quantity of electric charge, *V* is the volume of gas, *I(t)* is the current of different time, *t* is time, *M*_*0*_ is the value of gas flow.

**Fig 4:**
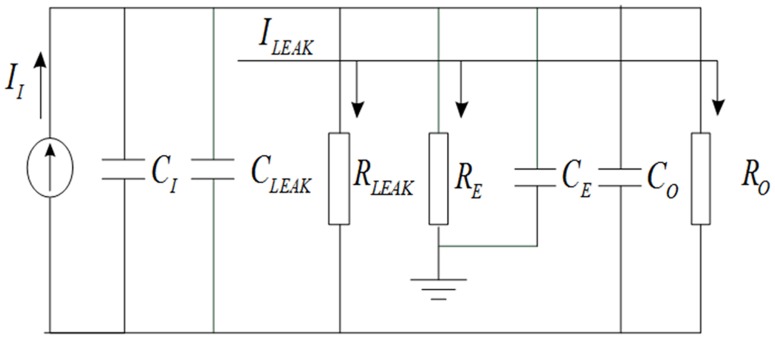
Diagram of equivalent circuit model.

Space charge measuring instrument of filter material and casing foil includes a capacitive sensor which can be equivalent to a current source for its lower magnitude of the space charge. As the space charge signal is very weak, the integrator circuit can convert electric charge to voltage (Q-V conversion). The weak signal charge is directly measured as the voltage signal. Sensors and Q-V conversion circuit equivalent model are shown in Fig 4.

### Wireless data acquisition node

**Fig 5:**
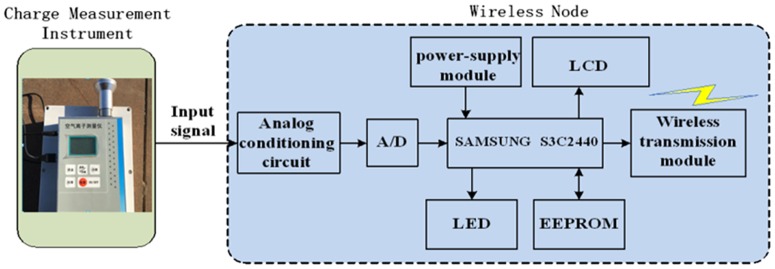
Block diagram of wireless data acquisition node.

Wireless data acquisition node is shown in Fig 5. The main function of wireless node is to receive and conduct the output of analog signal by charging measuring instrument and then to transmit the digital signal to the remote node.

The wireless node is connected with space charge sensor through the twisted-pair braid shielded cable to complete the signal transmission and power supply. The value of space charge can be directly obtained through the LCD screen in the wireless node. The core of the wireless node is the SAMSUNG S3C2440 embedded microcontroller and 2.4GHz Xbee Pro radio frequency (RF) module.

The signal conditioning circuit lifts the voltage from±1.5 v to 0–3.3 v. In order to make the signal suitable for AD conversion, the low pass filter circuit and impedance matching circuit are employed to amplify the signal. The high-precision operational amplifier with low offset voltage is used to reduce the fault rate of the signal conditioning circuit. The utilization of LCD can facilitate the view and record of data for operator. The power management unit supplies the LCD module, and can be switched to the different power modes when the wireless node acquires orders from the upper computer.

The 2.4GHz Xbee Pro radio frequency (RF) module is selected as the sensor node for wireless communication system. To finish RX/TX operation, the RF module needs to be operated under voltage level ranges from 2.5 to 3.5V and current level from 40mA to 50mA. The common transmit range for the RF module is about 1 km line-of-sight using 2.0 dB dipole antenna. Xbee Pro module in the wireless sensor node is utilized because of its convenience. The connections include the power and ground lines between the Din/Dout pins of RF module and the S3C2440 Rx/Tx pins.

**Fig 6:**
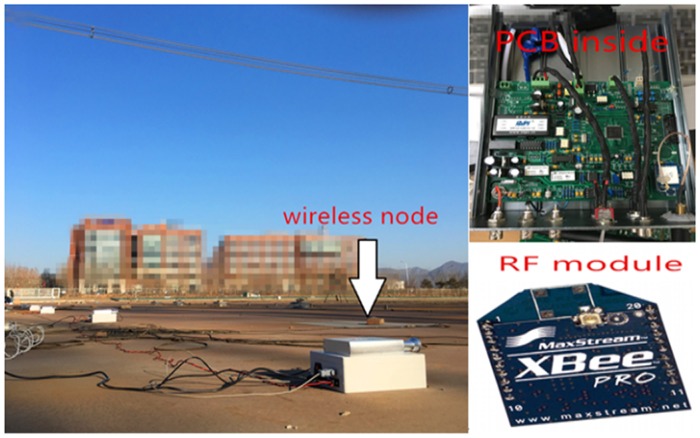
Picture of wireless node.

The wireless node receives the analog signal from space charge measuring instrument by the integrated AD convertor. The received data is processed by low pass filter. The output data of charge density is collected and then sent to the radio frequency module by RS232. The configuration of the wireless node is changeable. S3C2440 can be programmed to link with the space charge measuring instrument whose data is sent by the computer and deposited into the ROM. The proposed wireless node is shown in Fig 6.

### Wireless sensor network

**Fig 7:**
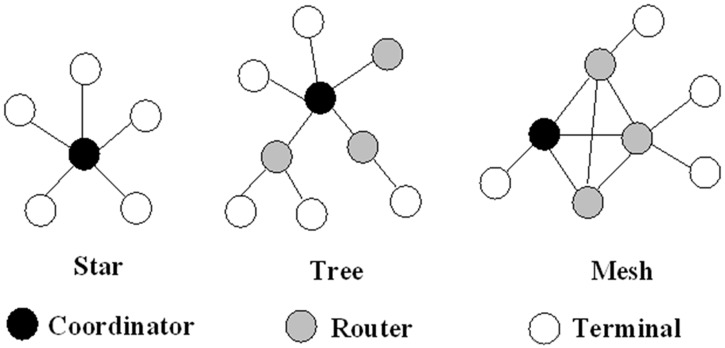
Topology of three kinds of network.

Zigbee technology supports three kinds of net space charge density measuring system and wireless node space charge density measuring system and wireless node work topology: star structure, cluster tree structure and mesh structure, as shown in Fig 7. The device object is divided into three different types: coordinator, router and node equipment. There is only one coordinator in an independent network, which is responsible for establishing the network, allowing the sub nodes to join, distributing the short address, and providing data router and security management services. Router is the relay station of network which allows other sub nodes to route through it. The node equipment can only send or receive data through a router or coordinator. In the Zigbee network, according to the different communication capabilities of the nodes, the network equipment can be divided into two kinds: Full Function Device (FFD) and Reduced Function Device (RFD). Compared with FFD, RFD circuit structure is more simple and the memory capacity is smaller. However, it cannot communicate directly between RFD and only serves as its parent node of the FFD communication. The memory capacity of FFD must be relatively large, which can provide the exchange of data. An FFD can communicate with other multiple FFD or RFD. Coordinator and router should be capable of relaying and storing data, because of which FFD is selected, while node equipment can be FFD or RFD.

The system consists of a coordinator and multiple node devices. Each node equipment can only be connected with coordinator to control the whole network. The cluster tree structure and mesh structure are all the way through the router. The router can communicate directly with the network and also provide multiple paths according to different routing strategies.

**Fig 8:**
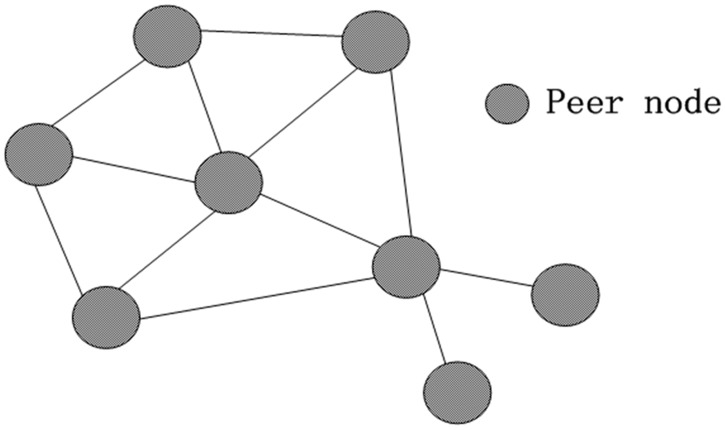
DigiMesh Topology of network.

Considering the complexity of actual environment and the requirements of transmission line, a Mesh network basing on DigiMesh network towards the topology of network (firmware version is XBP24-DM) is employed in present work. Fig 8 presents the DigiMesh topology of network.

In comparison to Mesh network, the function of DigiMesh network has been simplified. All the nodes are functionally equivalent, and all of the sub nodes can be used as the end of the main nodes. If there is a large area of sub nodes that can't work, the node which is closer to the main node will automatically assume the routing function in order to achieve the reliability of network for transmission lines under the extreme circumstances. In practice, only the target address of all sub nodes (L and DH) can set up the 64-bit physical address (SL and SH) to achieve the convergence and integration of data.

## Validation and Experiment

### Communication quality in complex environment

Complex environment is commonly involved under high-voltage transmission lines, various factors including electromagnetic interference, topographic relief, surface vegetation and weather change are prone to exerting impact on communication quality. Herein, enhanced performance of the whole system is anticipated to be realized by comprehensively investigating and solving the effect of external environmental factors. To date, lots of basic parameters have been employed to evaluate the performance of the present measurement system, the value of relative signal strength indicator (RSSI), which is capable of receiving data frames, is employed for analysis in present work. RSSI is the difference value between real received signal strength and optimum received power level, that is to say, higher absolute value of RSSI represents greater signal attenuation. RSSI value can be obtained when each data frame is received by Zigbee transceiver module. In present system, AT command was sent by host node, followed by the return of data frame, by which RSSI value is obtained, the structure is clearly listed in Table 1. AT Command denotes “DB” in Table 1 means the inquire of signal strength of last data frame, the obtained Command Data therefore is RSSI value.

**Table 1:** AD Command Response of Xbee Pro.

Frame Fields	Start Delimiter		Length		Frame Type	Frame ID
Example	0x7E		0x00	0x05	0x88	0x01
Frame Fields	AT Command		Command Status		Command Data	Checksum
Example	‘D’ = 0x44	‘B’ = 0x42	0x00		NULL	0xF0

The commonly used path loss model is for free space radio propagation, as shown in formula 2.

Lbf=32.44+10nlgf+10nlgD (2)

where *Lbf* (dB) is the path loss of radio signal with a distance of *D* (km), *f* is radio frequency (MHz), *n* is the path attenuation factor and commonly taken as 2–5. The signal intensity can be calculated using the following formula:

$$RSSI=P_{t}+G_{t}+G_{r}-L_{c}-Lbf.$$ (3)

where *P*_*t*_ is the power of the transmitted signal, *G*_*t*_ is for transmitting antenna gain, *G*_*r*_ is for receiving antenna gain, *L*_*C*_ is for line loss. In practical application environment, both radio propagation path loss and theoretical value would change with various factors including multipath, diffraction and obstacle. After making some transformation and simplification:

RSSI=b+k*20lgD (4)

Among them

$$b=P_{t}+G_{t}+G_{r}-L_{c}-32.44-10n\text{lg}f$$ (5.1)

k=−n/2 (5.2)

It is thus found that when *P*_*t*_, *G*_*t*_, *G*_*r*_, *L*_*c*_ are the same, *RSSI* value is related to the attenuation factor *n* and the transmission distance *D*.

**Fig 9:**
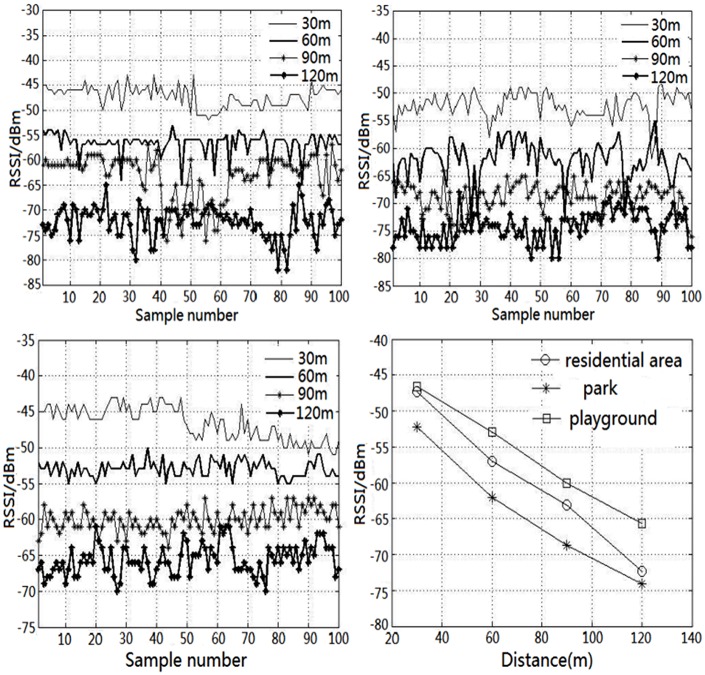
RSSI value of different places.

In consideration of the practical application in complex environment, three typical scenes for the experiment are chosen, including: residential area, park and playground. In each scenario, the test nodes are separately placed 30m, 60m, 90m and 120m away from the main node, and the three experimental results are shown in Fig 9.

For the same environment, the *k* and *b* in formula 5 can be considered as the same. As shown in Fig 9, with the increase of transmission distance, RSSI is gradually reduced. The results keep good consistency with the calculated results derived from formula 4.

The packet loss rates of the three experiments are shown in Table 2.

**Table 2:** Packet Loss Rate under Different Environment.

Different distances	Playground	Park	Residential area
30m	0.2%	1.8%	0.2%
60m	0.5%	3.9%	0.6%
90m	0.9%	10.2%	2.3%
120m	2.8%	30.3%	5.1%

Evidently, it can be deduced from Table 2 that when the distance is less than 60m, each kind of environment presents little data packet loss, while all environments demonstrate data packet loss when the transmission distance reaches 120m. Therefore, the distance between the points less than 60m is preferred, otherwise it will cause the waste of node resources. Furthermore, the distance between the points should not exceed 120m, otherwise it will reduce the network performance to impact the normal data transmission.

### Space charge density measurement

**Fig 10:**
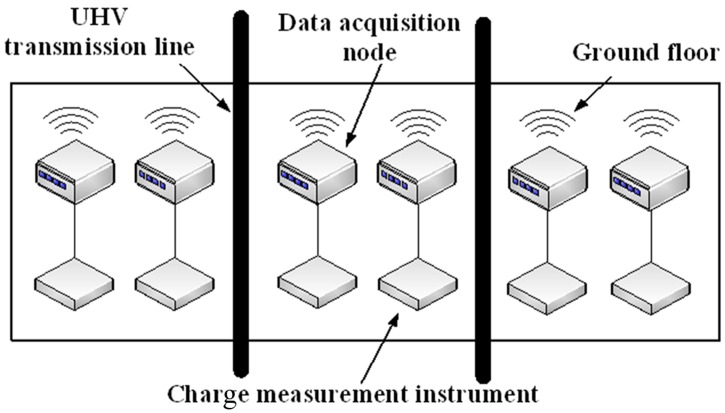
Measuring device placement.

The present measuring system has been carried out in Beijing, Hebei, Shandong and Tibet. In the case of Beijing test, the method of measuring device is shown in Fig 10.

**Fig 11:**
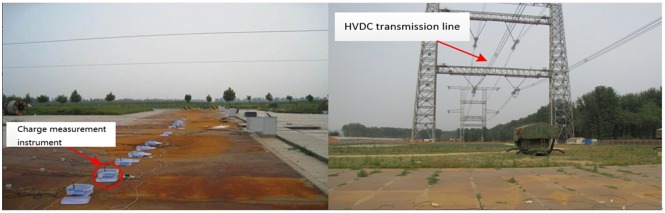
Picture of measurement system in HVDC test base.

Charge measuring instrument is vertical to the positive and negative pole conductor. Taking the centerline projection of negative pole conducting wire at the ground as the abscissa axis coordinate origin, the sensors in the outward extension of -10m, -8m, -7m, -6m, -5m, -4m, -3m, 0m, 3m, 4m, 5m, 6m, 7m and 8m are placed. To test the wireless sensor network system, the sensors are put under a double circuit experimental line in China Electric Power Research Institute in Beijing. The UHDV line is 150m long and the model of the UHDV line is LJG-240/40. Its distance from the ground floor is about 8m and the distance between two poles is 7m. Fig 11 gives the picture of measuring system used in HVDC test base.

**Fig 12:**
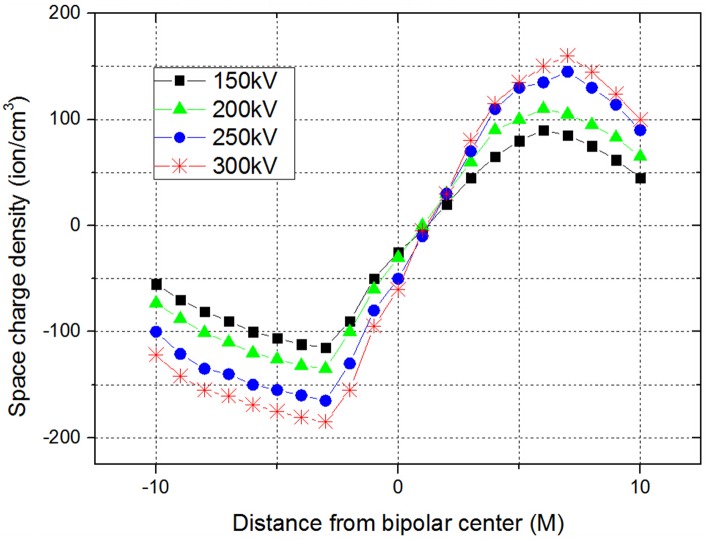
Experiment results of space charge density under different voltage levels.

The wire voltage of the positive pole was set up at 150kV, 200 kV, 250 kV and 300kV. Correspondingly, the wire voltage of the negative pole was set up at -150 kV, -200 kV, -250 kV and -300 kV. It is found that the measuring results are prone to be greatly influenced by environmental factors. The temperature is determined to be about 28 to 32 degree and relative humidity is 50%. The testing site is empty without other shelters. It was a good day with no wind. A lot of experimental data of the space charge is obtained under HVDC lines. A large number of experimental measurement data of the electric field is obtained under the double circuit experimental lines on the same tower. One of the experimental results of space charge density under different voltage levels is shown in Fig 12.

In Fig 12, for a fixed wire with different voltage levels, the absolute value of space charge density is greater when the distance between the ground and poles is shorter, which reaches the maximum value when the poles wire is near to the line. In the center of the positive and negative pole, the space charge density is almost zero. The space charge density of each measuring point under the negative pole is generally larger than that of the positive line. With the increase of voltage level, the space charge density of the ground under the transmission line is increased. The space charge is the superposition of the nominal space charge and ion current. Actually, the measurement of ion current will be affected by various weather conditions, such as humidity, wind speed, temperature, pressure, etc. Herein, the measured values will inevitably deviate from that of calculated ones to some extent.

## Discussion

Much efforts in past years have been devoted and the results suggested that space charge buildup under high voltage direct current (HVDC) transmission line is likely to be strongly affected by various factors, such as performance of insulators, psychological and physiological effects on human body, the former of which includes the distortion of electrical stress distribution due to space charge formation and accumulation, electrical treeing initiation, breakdown as well as insulation aging. The latter one is more important as it directly exerts impact on human body which has aroused wide public concern. Many studies show that the presence of space charge greatly influences the electric field distribution. Hence, it is of great importance to measure the space charge accurately and conveniently under HVDC transmission lines.

Space charge measurement is a reliable non-destructive method and has become a major concern of the electrical industry to investigate solid insulating material under high electric field. Space charge measurements provide operable means to detect magnitude, polarity and location of charge trapped in a dielectric. This shows that space charge measurement is a precious tool to evaluate the dielectric properties by comparing the tendency of material with accumulated charge. Many methods have been developed to probe space charge distribution and measure space charge density. Traditionally, using cable for space charge density monitoring system is very inconvenient for its large volume, low flexibility and weak self-organization. However, Wireless Sensor Network (WSN) can solve this problem. Specifically, space charge under HVDC transmission lines is measured at different positions through many wireless sensors. The whole measuring system is composed of three parts: signal sensing, data transmission and data processing. The components of space charge sensor include outer shielding shell, front filter, main filter, flow meter, differential pressure gauge, fan and measuring circuit. The actual testing results show that the measured values inevitably deviate from and calculated value, which is believed to be a result of environmental influence.

In short, compared with the traditional communication network, WSN demonstrate its advantages in small volume, high flexibility and strong self-organization, which is therefore more suitable for the construction of space charge distribution and density monitoring system as it has long distance and high mobility. Furthermore, a distributed wireless system is designed for collecting and monitoring the space charge density under HVDC transmission lines. This measurement system has been studied for years and has been used in Chinese state grid HVDC test base and power transmission projects. All the experimental results show that this measuring system can meet all the demands of the complex electromagnetic environment under HVDC transmission lines and can measure the space charge density accurately, flexibly, and stably.

## Conclusion

A distributed wireless system aims at measuring space charge density under HVDC transmission lines for accurate, flexible, and stable measurement of the space charge is developed in present work. The proposed system architecture is composed of a group of wireless nodes connected with charge measuring instrument and a base station. Zigbee module and ARM are used in the design of wireless nodes for the system. Compared with the traditional space charge monitoring system, the proposed system has advantages of strong extensibility and stability under HVDC transmission lines. The maximum number of testing points can reach 250, and all the experimental results prove that the proposed measurement system can be applied to collect the space charge density data in the complex electromagnetic environment.
